# Sentinel lymph node biopsy of the internal mammary chain in breast cancer

**DOI:** 10.1007/s10549-012-2086-5

**Published:** 2012-06-08

**Authors:** E. L. Postma, S. van Wieringen, M. G. Hobbelink, H. M. Verkooijen, H. J. G. D. van den Bongard, I. H. M. Borel Rinkes, A. J. Witkamp

**Affiliations:** 1 Department of Surgery, University Medical Centre Utrecht, Postbus 85500, 3508 GA Utrecht, The Netherlands; 2 Department of Imaging, University Medical Centre Utrecht, Postbus 85500, 3508 GA Utrecht, The Netherlands; 3 Department of Radiation Oncology, University Medical Centre Utrecht, Postbus 85500, 3508 GA Utrecht, The Netherlands; 4 Department of Surgery, University Medical Centre Utrecht, Heidelberglaan 100, 3584 CX Utrecht, The Netherlands

**Keywords:** Sentinel lymph node biopsy, Internal mammary chain, Breast cancer, Post-operative treatment

## Abstract

Routine removal of the internal mammary chain (IMC) sentinel node in breast cancer patients remains a subject of discussion. The aim of this study was to determine the impact of routinely performed IMC sentinel node biopsy on the systemic and locoregional treatments plan. All patients with biopsy proven breast cancer who underwent a sentinel node procedure between 2002 and 2011 were included in a prospective database. In cases of IMC drainage, successful exploration of the IMC (i.e., sentinel node removed) and surgical complications were registered. If the removed sentinel node contained malignant cells we determined if this altered the treatment plan when practising the current guidelines. In total, 119 of the 493 included patients showed IMC drainage on lymphoscintigraphy. Exploration of the IMC was performed in 107 (89 %) patients; in 86/107 (80 %) exploration was successful. In 14/107 patients (13 %) the IMC sentinel node was tumor positive. Macro and micro metastases were found in eight and six patients, respectively. In the group of patients who underwent surgical exploration of the IMC, systemic treatment was changed in none, radiotherapy treatment in 13/107 patients (11 %). Routine sentinel node biopsy of the IMC does not alter the systemic treatment. Radiotherapy treatment is altered in a small proportion of the patients; however, solid scientific evidence for this adjustment is lacking.

## Introduction

Sentinel lymph node (SLN) biopsy is a standard procedure for axillary assessment of patients presenting with clinically node-negative early breast cancer [1–4]. Depending on the technique of injecting the nanocolloid and the site of the tumor, extra axillary lymph drainage to the internal mammary chain (IMC) is found in up to 30 % of breast cancer patients [5–7]. There is however, no consensus on the added diagnostic or prognostic value of retrieving SLNs from the IMC when seen on pre-operative lymphoscintigraphy. Opponents point out that harvesting these nodes has no clinical relevance because tumor-positive IMC SLNs rarely influence adjuvant systemic treatment strategy and because there is no evidence supporting an effect of radiotherapy (RT) to the IMC [8, 9]. Besides, they state that the increased radiation dose that is administered to the cardiac and pulmonary tissue leads to increased morbidity and mortality. Proponents of routine IMC SLN biopsy advocate that the presence of lymph node metastases in the IMC is associated with a poorer prognosis in a small but substantial patient group and that these metastases should therefore be treated with appropriate systemic therapy and IMC RT [6, 7, 10–13]. As a reflection of this ongoing debate, Dutch national guidelines on the treatment of breast cancer do not recommend routine biopsy of the IMC SLNs. Adjuvant chemotherapeutic treatment and IMC RT is however, indicated when a tumor-positive IMC lymph node is found [14].

In this historical cohort study, we evaluated the impact of IMC sentinel node biopsy (which is routinely performed in our hospital) upon the systemic and locoregional treatment strategies.

## Methods

Between January 2002 and August 2011 all patients with biopsy proven cT1-2 cN0 invasive breast cancer underwent surgical treatment including sentinel node biopsy. All patients were included in a prospective database. Patient who did not show drainage on lymphoscintigraphy, who received neo-adjuvant systemic treatment, in whom the tumor was multicentric or those with recent surgery to the ipsilateral axilla or breast were excluded from our analysis.

### Sentinel node procedure

A mean dose of 120 MBq Tc-99m nanocolloid in a 0.5 cc physiological saline was administered through four peritumoral injections. A higher dose (370 MBq) was administered when the sentinel node procedure was performed according to a 2-day protocol. Early and late static images (anterior and lateral) were acquired with a single or dual head gamma camera 1 and 2 h post-injection. If no sentinel node was depicted, an extra series of late images was performed often after administration of additional TC-99m nanocolloid. The sentinel node was marked on the skin. In patients with non-palpable tumors the radiotracer was injected intratumorally guided by either ultrasound or stereotaxis, depending on the visibility of the tumor.

### Surgery

Lymphatic mapping procedure was performed the previous day or on the same day as surgery. In the operating theatre 1–2 ml Patent Blue was injected peritumorally. The sentinel node was identified with the aid of blue dye and the gamma probe. Axillary as well as IMC sentinel nodes were excised whenever possible.

### Histologic examination

All sentinel nodes were bisected if their size was >0.5 cm. Both parts were formalin fixed and step sections were made at 250 μm-intervals. H&E staining was performed. In addition immunohistochemical staining was performed if H&E staining proved negative. If metastases were present, they were classified as macro metastases, defined as a metastatic depot of >2 mm in size; as micro metastases, defined as a metastatic depot of 0.2–2 mm in size; or as isolated tumor cells, defined as a single tumor cell or a cluster of tumor cells of <0.2 mm in size [15].

### Outcome measures

For each patient, information on patient and tumor characteristics was gathered. We analyzed the lymphatic drainage pattern and determined the proportion of patients with IMC drainage. In this selection of patients, evaluation of the number of successful IMC sentinel node biopsies during surgery, intraoperative complications due to the attempt of harvesting the IMC sentinel node and the frequency of metastases in the IMC node was performed. We then analyzed the effect of the IMC sentinel node histology on the adjuvant treatment according to the most recent Dutch guideline on the treatment of breast cancer [14].

### Statistical analysis

Normally distributed continuous variables were presented as means (standard deviation) and compared with independent *t* tests. Not normally distributed data were presented as medians (range) and compared with the Mann–Whitney *U* test. Chi-square test was used to compare proportions. Differences were considered significant when *p* < 0.05.

## Results

**Fig. 1:**
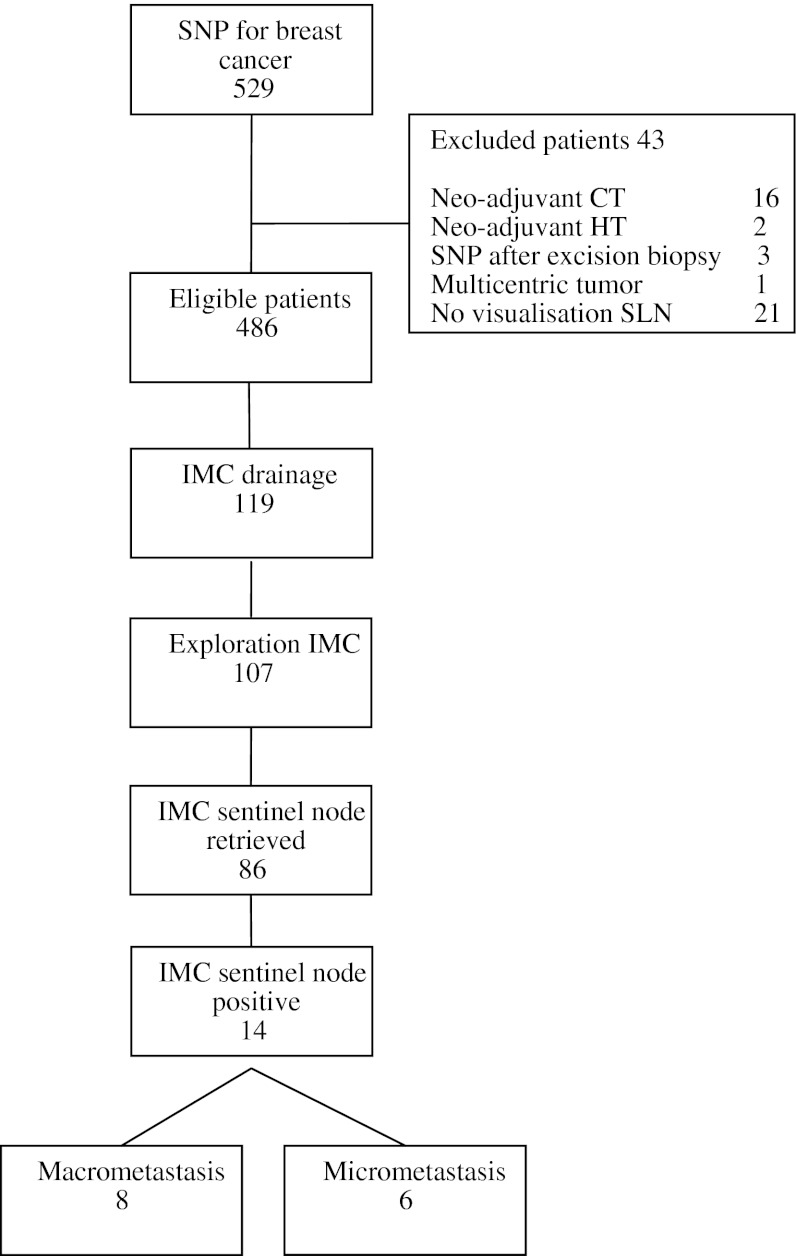
Flowchart sentinel lymph node procedures. *SNP* sentinel lymph node procedure, *IMC* internal mammary chain, *CT* chemotherapy, and *HT* hormonal therapy

In total, 486 patients who underwent 493 sentinel node procedures were included in this retrospective analysis (Fig. 1). Forty-three patients were excluded; 3 patients underwent a SNP after excisional biopsy, 18 patients received neo-adjuvant systemic treatment, in 1 patient the tumor was multicentric and 21 patients did not show drainage on lymphoscintigraphic imaging. Lymphoscintigraphic imaging showed axillary drainage in 479 (99 %) patients. An IMC drainage pattern was seen in 119 of 486 (24 %) patients; 112 with concomitant drainage to the axillary nodes and 7 with drainage to the IMC exclusively. IMC drainage was associated with a smaller tumor size, non-palpability and a medial localization of the tumor (Table 1). Biopsy of the axillary SN was successful in 478/481 (99 %) and contained metastases in 164/478 (34 %) of the cases. Biopsy of the IM SLN was successful in 86/119 (72 %) of the patients with drainage to the IMC. In 12 patients exploration of the IMC region was not attempted because the radioactivity count was considered too low in this region intraoperatively. In the remaining 21 patients, exploration of the parasternal region was attempted, but unsuccessful. The IMC sentinel node contained metastases in 14 patients; histopathologic examination showed macrometastatic disease in eight patients and micrometastatic disease in six patients.

**Table 1:** Patient and tumor characteristics

	All patients^a^ *N* = 486	IMC drainage *N* = 119	No IMC drainage *N* = 367	*p* value
Mean age (SD)	58 (22–86)	56 (53–81)	59 (22–86)	0.101
Median tumor size in cm (SD)	1.9 (1.2)	1.7 (0.9)	2 .0 (1.2)	0.044
Tumor localization				
Central	54 (11 %)	14 (12 %)	40 (11 %)	<0.001
ULQ	218 (45 %)	27 (22 %)	191 (52 %)	
UMQ	68 (14 %)	29 (24 %)	39 (11 %)	
LLQ	52 (11 %	16 (13 %)	36 (10 %)	
LMQ	48 (10 %)	18 (15 %)	30 (8 %)	
Cranial	19 (4 %)	7 (6 %)	12 (3 %)	
Caudal	5 (1 %)	3 (3 %)	2 (1 %)	
Medial	5 (1 %)	3 (3 %)	2 (1 %)	
Lateral	17 (3 %)	2 (2 %)	16 (4 %)	
B & R grading				
1	107 (22 %)	22 (18 %)	85 (23 %)	0.283
2	179 (37 %)	50 (42 %)	129 (35 %)	
3	149 (31 %)	38 (32 %)	111 (30 %)	
Unknown	51 (10 %)	9 (8 %)	42 (11 %)	
Tumor histology				
IDC	382 (78 %)	94 (77 %)	288 (76 %)	0.824
ILC	38 (8 %)	7 (6 %)	30 (8 %)	
IDLC	51 (10 %)	12 (10 %)	39 (11 %)	
Other	27 (6 %)	5 (3 %)	10 (2 %)	
Axillary involvement				
N 0	322 (66 %)	87 (73 %)	235 (64 %)	0.069
N +	164 (34 %)	32 (27 %)	132 (36 %)	
ER				
Positive	405 (83 %)	94 (80 %)	311 (85 %)	0.385
Negative	60 (12 %)	17 (14 %)	43 (12 %)	
Missing	21 (5 %)	8 (3 %)	13 (4 %)	
PR				
Positive	328 (67 %)	77 (65 %)	251 (68 %)	0.757
Negative	137 (28 %)	34 (29 %)	103 (28 %)	
Missing	21 (4 %)	8 (7 %)	13 (5 %)	
Palpable				
Yes	308 (63 %)	66 (58 %)	242 (66 %)	0.027
No	174 (36 %)	53 (42 %)	121 (33 %)	
Missing	4 (1 %)	0 (0 %)	4(1 %)	
Bilateral SNP	7 (1 %)	0 (0 %)	7(2 %)	0.113

*ULQ* upper lateral quadrant, *UMQ* upper medial quadrant, *LLQ* lower lateral quadrant, and *LMQ* lateral medial quadrant

^a^In patients with bilateral disease, data of the first diagnosed tumor was used for analysis

Of the 14 patients with IM SLN metastases 7 (50 %) were presented with concomitant axillary lymph node involvement. Conversely 7/165 patients (2 %) with axillary metastases, showed IMC involvement.

### IMC sentinel node biopsy

Surgical exploration of the IMC was performed in 107 patients. In 94 patients information regarding the incision for IMC exploration was reported; an extra incision was necessary in 86 patients and in eight patients exploration was performed using the lumpectomy or mastectomy incision. Serious complications were reported in 3/107 patients (3 %); in two patients re-exploration was necessary due to post-operative bleeding, one patient developed a haematothorax after surgery. In six patients minor injury to the pleura during surgery was reported; all patients were treated conservatively. Intraoperative bleeding from the internal mammary artery occurred in four patients, but was stopped successfully.

### Change in systemic treatment strategy

Based on unfavorable primary tumor characteristics or involvement of the axillary SLN status, hormonal treatment was indicated in 13 patients and chemotherapy in 11 patients. The IMC sentinel node histology did not affect the adjuvant treatment in any of the patients (Table 2).

**Table 2:** Patients with IMC lymph node metastases (*n* = 14)

Pt	IMC SLN	Age	Size	BR	HR	Ax+	Ind CT	Ind HT	Ind CT	Ind LRRT	Change
1	1 macro	73	1.9	III	E+ P+	0	–	+	–	No	LRRT
2	1 macro	71	1.2	II	E+ P−	1	–	+	–	No	LRRT
3	1 micro	73	2.5	II	E+ P+	1	–	+	–	No	LRRT
4	1 macro	32	1.3	NR	E+ P+	0	+	+	PTC/age	No	LRRT
5	1 macro	50	2.1	III	E− P−	7	+	–	PTC + AX	Yes	No
6	1 macro	65	2.5	III	E+ P−	2	+	+	PTC + AX	No	LRRT
7	1 micro	57	2.6	II	E+ P+	1	+	+	PTC + AX	No	LRRT
8	1 macro	47	0.5	II	E+ P+	1	+	+	AX	No	LRRT
9	2 micro	64	1.7	III	E+ P+	0	+	+	PTC	No	LRRT
10	1 macro	57	1.5	II	E+ P+	0	+	+	PTC	No	LRRT
11	2 macro	61	1.5	III	E+ P+	0	+	+	PTC	No	LRRT
12	1 micro	46	2.0	III	E+ P+	0	+	+	PTC	No	LRRT
13	1 micro	50	1.3	III	E+ P+	0	+	+	PTC	No	LRRT
14	1 micro	36	3.5	III	E+ P+	1	+	+	Size + AX	No	LRRT

Patient characteristics, primary tumor characteristics and therapeutic consequences when practising current guidelines

*IMC SLN* internal mammary chain sentinel lymph node (histologic outcome), *Size* tumor size in cm, *BR* Bloom and Richardson grade, *HR* hormone receptor status, *E±* estrogen receptor positive/negative, *P±* progesterone receptor positive/negative, *Ax+* axillary lymph node metastases, *CT* chemotherapy, and *LRRT* locoregional radiotherapy

### Change in radiotherapy treatment

A change in the RT plan was seen in 13 patients due to a tumor-positive IMC node. IMC and medial periclavicular lymph nodes were irradiated in these patients. One patient presented with seven positive axillary lymph nodes. As such, she already had an indication for locoregional radiotherapy of the parasternal and medial periclavicular area.

## Discussion

In the group of patients studied, lymphatic drainage pattern to the IMC was seen in 24 %. The majority (72 %) of explorations of the IMC was successful. Intra and post-operative complications related to this procedure were reported in 13/107 (12 %) of the patients. Histologic examination of the retrieved IMC sentinel nodes revealed metastases in 17 % of the patients. This rate is confirmed with other studies, considering the use of peritumoral or intratumoral injection of the radiotracer [6, 7, 9, 11]. The proportion of patients with IMC metastases and concomitant axillary metastases (50 %) is however, low compared to results reported by others [5–7, 11, 12, 16]. According to the current guidelines, exploration of the IMC leads to adjustment of the systemic treatment in none of the patients. However, adjustment of RT was seen frequently (11 %).

Several studies reported that prognosis of patients with medially located tumors is inferior to that of patients with laterally located tumors [17, 18]. Since it is known that medial tumors more often drain to the IMC, the rationale for harvesting IMC SLNs is the assumption that the poorer prognosis of patients with medially located tumors is a result of understaging of IMC lymph node metastases with the consequence of omitting adjuvant treatment in this patient group [11, 19, 20]. As sentinel lymph node biopsy of the IMC leads to a greater degree of staging accuracy, it provides a guidance for a more specific tailored therapy. Studies evaluating the effect of the IMC SLN biopsy on the treatment strategy in patients with an IMC drainage pattern report change of treatment in 2–9 % [5, 7, 11, 19]. Since adjuvant systemic treatment in this small but substantial patient group is likely to improve prognosis, authors of these studies recommend routine biopsy of IMC SLN’s.

Despite these considerations, the clinical value of IMC SLN biopsy remains heavily debated. Since unfavorable primary tumor characteristics solely have become a sufficient indication for adjuvant systemic treatment, the proportion of patients with an indication for systemic treatment has increased substantially. Moreover, solely IMC metastases changing the N0 to an N+ status are rare. Rates of 2–8 % have been reported by others [6, 7, 11, 16]. We observed seven patients with IMC metastases without axillary involvement, representing only 1 % of our study population. Although biopsy of the IMC SN leads to upstaging in these patients, systemic treatment was not affected as all patients already would have received systemic treatment based on unfavorable tumor characteristics. Consequently, the clinical value of determining the IMC SLN status with regard to the systemic treatment has decreased. Our study confirms this, as the systemic treatment plan was not changed in any of the patients.

Regarding the RT adjustments; for every nine patients in whom biopsy of the IMC SLN was attempted, one patient was identified eligible for additional (locoregional) RT treatment. Straightforward evidence of the value of RT in terms of improving long-term prognosis is however, lacking. Romestaing et al. [21] randomized patients to RT versus no RT of the IMC after surgery. In total, 1,334 early stage breast cancer patients with node-positive disease (75 %) and/or medially located tumors that underwent mastectomy and RT to the chest wall, axilla and periclavicular area were enrolled. No significant difference was found in 10 year overall survival. As the IMC lymph nodes were not pathologically evaluated, it is likely that only a small proportion of these patients have actual tumor-positive IMC lymph nodes. This means that the study is possibly underpowered for showing a significant difference in the recurrence or survival rate. Another randomized comparison was carried out by Kaija et al. [22] evaluating the (dis)advantages of IMC RT. Radiation of the IMC did not lead to an increase in complications. No difference in disease recurrence was found; however, follow-up time was too short to be conclusive.

Results of an ongoing study (EORTC trial 22922) investigating the role of internal mammary and medial supraclavicular lymph node chain irradiation in I–III stage breast cancer are awaited [23]. However, inclusion criteria (high-risk patients/no histopathology of the IMC SLN) mean that this study will not adequately address the question of the additional value of IMC irradiation in patients with a tumor-positive IMC SLN.

Several trials evaluated the effect of locoregional RT in high-risk patients and showed that locoregional RT leads to a substantial improvement in locoregional control and a less substantial, but still significant improvement in long-term survival [24–27] (Table 3). This indicates that in widespread locoregional disease chemotherapy alone inadequately eliminates metastatic disease and an additional therapeutic effect is achieved by locoregional therapy. In these studies however, patients were treated with outdated chemotherapy regimens and underwent incomplete axillary lymph node dissection, rendering these results not applicable to current breast cancer patients. The ongoing NCIC-CTG MA-20 trial [28] reports on 1,832 high-risk node-negative and node-positive patients that underwent breast conserving surgery and systemic therapy between 2000 and 2007. Patients were randomly allocated to undergo regional node irradiation radiotherapy (RNI) or not. Interim analysis, after 5 years of follow-up shows that additional RNI reduces the risk of locoregional and distant recurrences, and improves the disease-free survival with a trend toward improved overall survival.

**Table 3:** Overview of studies evaluating the effect of locoregional RT in high-risk patients

Study	*N*	Patients	FU	ST	Outcome	GRM + RT	GRM	*p* value
Ragaz	318	N+	20	CMF	Overall survival	47 %	37 %	0.03
					Locoreg. RFS	90 %	74 %	0.002
Overgaard^a^	1,375	SII/III	10	Tam	Locoreg. recurrence	8 %	35 %	<0.001
					Overall survival	45 %	36 %	0.03
Overgaard^a^	1,708	SII/III	10	CMF	Locoreg. recurrence	9 %	32 %	<0.001
					Overall survival	54 %	45 %	<0.001
Clarke^b^	1,428	N0	5	Var.	Locoreg.	2.3 %	6.3 %	2*p* = 0.0002
	8,505	N+	5	Var.	recurrence	5.8 %	22.8 %	2*p* = 0.0002
	1,428	N0	15	Var.	Breast cancer	31 %	28 %	NS
	8,505	N+	15	Var.	mortality	55 %	60 %	2*p* = 0.0002

*FU* follow-up (years), *ST* systemic therapy, *GRM* *+* *RT* modified radical mastectomy + radiotherapy, *GRM* modified radical mastectomy, *var* various, *N+* node-positive disease, *N0* node-negative disease, *SII/III* stage II/III breast cancer, *RFS* recurrence free survival, *Tam* tamoxifen, *CMF* cyclophosphamide, methotrexate, 5-FU, *NS* not significant

^a^Incomplete axillary lymph node dissection: a median number of 7 lymph nodes were removed

^b^Meta-analyses in which the studies by Overgaard were included

Consideration should be given to the fact that in our study 6 of the 14 IMC SLN-positive patients presented with micrometastatic disease only. As the prognostic value of this finding is still questionable, it is unlikely that radiotherapeutic treatment in case of micrometastatic disease will result in a significantly improved prognosis [29, 30].

Irradiation of the IMC is accompanied by extra morbidity. Besides a marginally increased risk of secondary malignancy and radiation-induced lung injury, cardiac toxicity is a well known side effect of radiotherapy. Some studies show a significant increase in non-breast cancer mortality and long-term mortality from heart disease in breast cancer patients who underwent RT [31, 32]. Although modern CT-based RT planning techniques have proven to significantly decrease the irradiated heart dose and volume, long-term decreased cardiotoxicity has not yet been demonstrated. The fact that more aggressive systemic therapy regimens (including cardiotoxic agents) are frequently used nowadays and the long-term effect of RT in combination with these agents is not yet known, should also be considered. In our opinion, evidence of the additional value of IMC irradiation is not sufficient enough to legitimate the accompanying toxicity. Surgically staging by exploration of the IMC could be omitted by improving pre-operative staging. FDG PET/CT is shown to be a valuable instrument besides conventional modalities for the detection of extra-axillary lymph node macro metastases, also in regions that cannot be evaluated by ultrasound [33].

Exploration of the IMC is an extra procedure that carries an additional risk of intra and post-operative complications and a less satisfactory cosmetic outcome. Since the adjustment rate of systemic treatment based on the finding of this procedure is minimal and there is no sufficient ground for adjustment of RT, we believe that biopsy of the IMC SLN should not be performed routinely. As FDG PET/CT is of additional value for pre-operative staging and thus for selection of patients for RT, we advocate performing this procedure in high-risk patients (medially located tumor or N+).
